# Impact of Platelet Reactivity in ACS Patients on Clinical Outcomes with Triple Antithrombotic Therapy

**DOI:** 10.3390/jcm10081565

**Published:** 2021-04-08

**Authors:** Julia Gruttemeier, Yves Cottin, Hermann Yao, Emmanuel De Maistre, Maud Maza, Laurent Bonello, Marc Laine, Noemie Resseguier, Marianne Zeller, Laurence Camoin-Jau, Franck Paganelli

**Affiliations:** 1 Department of Cardiology, CHU Dijon Bourgogne, 21000 Dijon, France; julia.gruttemeier@sfr.fr (J.G.); yves.cottin@chu-dijon.fr (Y.C.); hermannyao@gmail.com (H.Y.); maud.maza@chu-dijon.fr (M.M.); 2 Department of Biological Haematology, CHU Dijon Bourgogne, 21000 Dijon, France; emmanuel.demaistre@chu-dijon.fr; 3 Department of Cardiology, ARCHANTEC, School of Medicine, Aix Marseille University, 13007 Marseille, France; laurent.bonello@ap-hm.fr (L.B.); marc.laine@aphm.fr (M.L.); 4 Public Health, Chronic Diseases and Quality of Life—Research Unit, Aix-Marseille University, 13007 Marseille, France; noemie.RESSEGUIER@univ-amu.fr; 5 Team PEC2, EA 7460, Department of Health Sciences, University of Burgundy Franche Comté, 21000 Dijon, France; marianne.zeller@u-bourgogne.fr; 6 Department of Biological Haematology, Assistance Publique Hôpitaux de Marseille, Timone Hospital, 13007 Marseille, France; laurence.camoin@ap-hm.fr

**Keywords:** acute coronary syndrome, triple antithrombotic therapy, VASP index, platelet reactivity, clopidogrel

## Abstract

Optimal antithrombotic therapy after percutaneous coronary intervention (PCI) in patients on oral anticoagulants (OAC) remains a clinical conundrum. In fact, combining an OAC with dual antiplatelet therapy (triple antithrombotic therapy, TAT) increases the risk of bleeding. Clopidogrel is the only thienopyridine recommended in TAT patients. Whether its response plays a relevant role in this setting remains uncertain. We aimed to evaluate the level of platelet reactivity inhibition (PRI) achieved by oral TAT in Acute Coronary Syndrome (ACS) patients undergoing PCI and its relationship with outcomes. We performed a multicenter prospective observational study and assessed PRI by vasodilator-stimulated phosphoprotein (VASP) index following a loading dose of clopidogrel. The primary endpoint was the incidence of major adverse cerebral or cardiovascular events (MACCE) at six months based on High on Treatment Platelet Reactivity (HTPR, VASP > 50%). The secondary endpoint was the incidence of bleeding at six months based on Low on Treatment Platelet Reactivity (LTPR, VASP < 16%). 491 patients were followed up for six months: 7.7% experienced MACCE and 17.3% experienced bleeding. There was no significant relationship between HTPR and MACCE, neither between LTPR and bleeding. Vitamin-K antagonist (VKA) treatment was associated with more MACCE and bleeding events, and the majority of events occurred within the first months. VASP index failed to predict outcomes in post-ACS patients with TAT. We confirm that direct acting OAC should be prioritized over VKA in TAT regimen.

## 1. Introduction

Triple antithrombotic therapy (TAT) (combination of oral anticoagulation, OAC, and dual antiplatelet therapy, DAPT) in the setting of acute coronary syndrome (ACS) is increasingly prescribed because of frequent associated co-morbidities [1]. These patients receive DAPT for the prevention of thrombotic or ischemic events, and oral OAC is recommended in patients with atrial fibrillation (AF), mechanical heart valves and venous thromboembolism [2,3,4,5]. Historically, patients who required OAC were systematically excluded from randomized controlled studies (RCT). Data from registries have shown that TAT was effective in reducing ischemic events, but with significantly increased risk of bleeding [3,6,7]. As the only thienopyridine recommended in recent guidelines for the TAT regimen, clopidogrel has been shown through platelet function assessment test (PFT) to have a high degree of inter-individual variability for platelet reactivity inhibition (PRI) [8]. High on-treatment platelet reactivity (HTPR) is associated with ischemic events and low on-treatment platelet reactivity (LTPR) is associated with bleeding [8,9]. Thus, the use of clopidogrel and individual variability between individuals (such as obesity, impaired kidney function) may make it difficult to implement TAT [9]. To address this issue, we aimed to investigate the inter-individual variability for PRI in ACS patients undergoing percutaneous coronary intervention (PCI) and receiving TAT regimen in order to identify sub-groups at higher risk populations for bleeding or ischemic events regarding HTPR or LTPR status.

## 2. Materials and Methods

### 2.1. Study Design

We conducted a multicenter observational prospective study from January 2018 to December 2019. We selected patients aged 18–90 years, with preexisting indication for OAC, admitted for ACS and who underwent PCI with stent implantation [3]. Patients receiving TAT regimen including clopidogrel for less than 48 h, patients who have been treated with ticagrelor or prasugrel for at least 24 h prior to PRI evaluation, and patients unavailable for PRI evaluation (holidays, weekends, etc.) were not considered in our study. Exclusion criteria were: history of intracranial bleeding, cardiogenic shock, contraindication for aspirin or clopidogrel in the previous six months, thrombocytopenia, major bleeding in the past month, pregnancy, concomitant severe illness with expected survival < 1 month, surgery within one month, liver failure. All patients undergoing PCI gave an informed consent before inclusion in the study. Patients were followed up for six months. The protocol was approved by local ethic committees (CPP “comité de protection des personnes” and ANSM “agence nationale de sécurité du médicament et des produits de santé”) and in accordance with the declaration of Helsinki [Prospective Real-world Registry Describing Treatment Regimens (PRAETORIAN) NCT03942913].

### 2.2. Periprocedural Antithrombotic Management

During PCI, additional low-dose of parenteral anticoagulation (bolus of unfractionated heparin 100 IU/kg UHF) was administered in all dabigatran-treated patients [10]. In other cases, PCI was performed without interruption of OAC and without additional anticoagulation, in accordance with the guidelines [3]. Additional UFH should be administered as per usual practice to support PCI, at standard dose (70 to 100 U/Kg) in patients receiving DOAC (direct oral anticoagulant) and reduced dose (30 to 50 U/Kg) in case of vitamin-K antagonist (VKA) therapy [3]. All patients received a bolus of 250 mg aspirin i.v. and a loading dose of 600 mg clopidogrel just before the PCI followed by daily DAPT regimen. In patients already on OAC, continuation of treatment with the same agent after PCI was encouraged, particularly if there was good compliance, good control of international normalized ratio (INR), and in patients free of ischemic and hemorrhagic events.

### 2.3. Risk Score, International Normalized Ratio, Dosing Regimen of a DOAC for Combination Therapy in TAT

CHA2DS2VASc scores reflect the risk of stroke among patients with AF who are not receiving anticoagulant therapy. HAS-BLED scores reflect the risk of bleeding among patients with AF who are receiving anticoagulant therapy [3]. For all patients who received VKA, the predicted INR was set between 2 and 2.5 [3]. Consistent with a recent review [3], DOAC was prescribed at full stroke prevention doses guidelines for AF (150 mg bid for Dabigatran, 5 mg bid for Apixaban). If rivaroxaban was used, the 15 mg once-daily dose (e.g., rather than the 20 mg dose tested in trials) was considered a reasonable alternative [3]. Because of DOAC short half-life and limited drug-drug interactions, blood level tests were not needed. In some situations, the issue of blood testing for DOAC levels arises but there is a consensus suggesting that it would not be an appropriate tool for the management of TAT patients [3].

### 2.4. Platelet Reactivity Measurements

PRI Measurements has been previously described. Blood samples were drawn at least 6 h and within 24 h after clopidogrel loading dose [11]. Briefly, a citrated blood sample was incubated with prostaglandin E1 (PGE1) or with PGE1 and ADP 10 μmol/L for 10 min and fixed with paraformaldehyde, after which the platelets were permeabilized with nonionic detergent. Analyses were performed on an EPICS XL-MCL flow cytometer (Beckman Coultronics, Margency, France). HTPR was defined as a VASP index ≥ 50% and LTPR as a VASP index < 16% [12,13].

### 2.5. Outcomes

The primary efficacy endpoint was major adverse cardiac or cerebrovascular events (MACCE) at six months (composite of all-cause mortality, non-procedural myocardial infarction, any urgent coronary revascularization, and ischemic stroke and/or extracranial thromboembolism). Deaths from vascular disease were defined as cardiovascular or cerebrovascular death and any death with unknown cause. New myocardial infarction and stroke have been previously defined and described [14,15]. Systemic embolism was diagnosed as acute arterial obstruction of the limbs or any organ and confirmed by angiography.

The secondary safety end point at six months was the occurrence of clinically significant bleeding (major bleeding, mild or moderate bleeding) according to Thrombolysis in Myocardial Infarction (TIMI) criteria or bleeding requiring medical attention [16].

### 2.6. Data Collection

Follow-up data after discharge was obtained during routine scheduled outpatient visits at one month and during telephone calls to patients every three months according to the clinical evolution. Standardized questions were used to assess bleeding episodes, thrombotic events and use of medications.

### 2.7. Patient and Public Involvement

For each patient, the investigator presented the advantages and disadvantages of the medical therapy and discussed the benefits of his participation to this study. Then, a consent form was signed in the presence of the investigator.

### 2.8. Statistical Analyses

Continuous data are expressed as mean (standard deviation) or median (interquartile range), and categorical data as numbers and percentages. Characteristics of the patients were described and compared according to the VASP index (cut-off: 50% or 16%). Statistical comparisons between groups used Student’s *t*-tests or Mann–Whitney tests for continuous variables, and chi-square test or Fisher exact test for categorical variables. The Cox regression model was used to estimate hazard ratios and their 95% confidence intervals. We used RStudio software version 1.2.5019 (2009–2019 RStudio, Inc., Boston, MA, USA). *p* values < 0.05 were considered significant.

## 3. Results

### 3.1. Population

**Figure 1:**
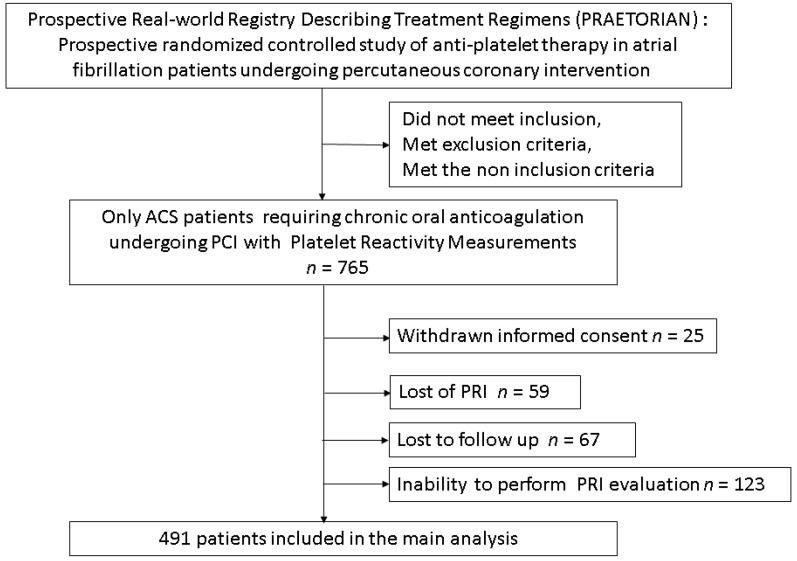
Flow-chart. ACS: acute coronary syndrome; PCI: percutaneous coronary intervention; PRI: platelet reactivity inhibition.

From PRAETORIAN registry, 765 consecutive patients receiving long-term OAC and PCI were assessed for eligibility. Withdrawn informed consent was found in 25 patients, 67 patients were lost to follow-up, PRI was not performed in 182 patients and these were excluded from the statistical analyses. A total of 491 patients were studied with a follow-up of six months (Figure 1). Table 1 and Table 2 summarizes the characteristics of the population. Of the 491 patients included, 72% were male and the median age was 78 years. Two hundred and seven patients (42.2%) had a history of CAD, and 99 had a history of bleeding (20%) including cerebral (0.8%) and gastrointestinal (7.5%) hemorrhages. The median CHA_2_DS_2_VASc score was 4.72 (4–6) and the median HAS-BLED score was 2.52 (2, 3). All patients were hospitalized for ACS, in most cases of NSTEMI; 99% of patients underwent PCI with stent implantation. Indication for OAC was mainly AF (75.7%), including 84.3% with prior history of AF and 15.7% with new-onset AF. Oral anticoagulant was VKA in 201 patients (41%), and DOAC in 290 patients (22.8% rivaroxaban, 22% apixaban and 14.2%, dabigatran). The median PRI score was 49% (25–69), but 250 patients (50.9%) had a score > 50%, reflecting poor response to clopidogrel therapy. LTPR was found in 39 patients (7.9%). In addition, the mean length of triple therapy was 3.51 months, which is in accordance with the recommendations at the time of the start of the study.

### 3.2. Major Adverse Cerebral and Cardiovascular Events at 6 Months

At six months follow-up, 38 patients experienced MACCE (7.7%). Sixteen had a recurrence of ACS (3.6%) and 18 died (3.7%). Two patients had an ischemic stroke (0.5%). Ischemic events were more frequent in the VKA group (Table 2).

### 3.3. Bleedings Events at Six Months

At six months, a TIMI bleeding event occurred in 85 patients (17.3%) receiving TAT regimen. Main bleedings were gastro-intestinal (44.7%) and 49% occurred in the VKA group. There were six major bleeds, 40 moderate, and 39 mild (Table 2). The effects of the different DOAC on ischemic and bleeding events are detailed in Table 2. No medical history or cardiovascular risk could be linked to the onset of bleeding or MACCE. The cumulative incidence of MACCE and bleeding at six months was highest among patients who had been assigned to receive VKA and lowest among those assigned to receive apixaban.

Based on the increasing evidence suggesting an association between HTPR and ischemic events, and between LTPR and bleeding events, we performed a further analysis on the occurrence of MACCE and bleedings regarding HTPR or LTPR status [11].

### 3.4. Bleeding and MACCE on HTPR or LTPR Group

Of all patients, 250 (51%) had a HTPR (VASP > 50%) with a high proportion of diabetics, and a higher prescription rate of DOAC (Table 3).

No difference was found in the HTPR group regarding MACCE, but conversely with a higher number of bleeding events (Table 4).

Only 8% (*N* = 39) had a LTPR with no increase in bleeding events. The characteristics of the patients at baseline were well balanced among the two groups except for more prescription of VKA agent in VASP > 16% group (Table 5 and Table 6).

## 4. Discussion

Our results provide the largest dataset of PRI in ACS patients undergoing PCI and receiving TAT. Our study failed to demonstrate that VASP assays could predict the clinical outcome of patients in this population. The differences between the two groups (HTPR, LTPR or not) regarding events were not statistically significant but this observation needs to be confirmed using a larger study population. The results were surprising, as it is known that HTPR is associated with ischemic events and LTPR is associated with bleeding [12,13].

### 4.1. PRI and MACCE or Bleedings at 6 Months

Several reasons may explain why PRI assessment did not predict the occurrence of MACCE or bleeding at six months in our study. First, data on cutoff values for VASP index in a setting of TAT regimen are limited, and it may well be that cutoff values that best determine patients risk for ischemia or bleeding may differ in this cohort compared with values obtained in patients with antiplatelet treatment alone [17]. Another possible reason for the negative results may be the assay used for the determination of PRI [18,19]. Danese et al. assessed the relationships between the pharmacokinetic and pharmacodynamic properties of clopidogrel, with the aim of identifying which is the best PFT [20]. The VASP assay was strongly influenced by the peak concentration of clopidogrel active metabolite. Timely performed VASP-P test may be the best marker for clopidogrel bioavailability and seems to be of potential usefulness in clinical studies aimed to identify the thrombotic and hemorrhagic risk associated with clopidogrel administration. Conversely, for Helten et al. multiplate impedance aggregometry could possibly be the best assay for antiplatelet medication tailoring at present [21]. In 2019, key opinions leaders summarized the updated expert consensus recommendations for the selective use of PFT in patients undergoing PCI [22]. Patients receiving OAC and antiplatelet treatment were described as an area of unmet need [19,20,21,22,23]. The only study analyzing PRI via VASP assays in patients on TAT regimen was conducted by Hu et al. [24]. In this study, 503 patients receiving TAT were randomized into two groups: the first group received dose titration of clopidogrel based on PRI, and the second group received a standard dose of 75 mg of clopidogrel. The results showed that the group receiving adjusted therapy based on PRI had significantly less MACCE at one year (2.5% vs. 5.0%, *p* = 0.02) without an increase in major bleeding (3.0% vs. 2.8%). However, these patients had a significantly higher rate of minor bleeding at 1 year than patients in the standard group (15.3% vs. 9.7%, *p* = 0.03).

### 4.2. VASP and TAT Regimen

To the best of our knowledge, this is the first prospective study assessing VASP assay in patients with TAT regimen. Compared with our study, Hu et al.’s population had a higher rate of MACCE (14.5% of patients) because their endpoints concerned target vessel revascularization (TVR) [22]. TVR is not influenced by antithrombotic regimen and it was not used in RCT- AF PCI patients [3]. Patients were younger (respectively, 63 years versus 80 years) with a lower CHA2DS2VASc score (3.7 versus 4.72 in our study). This study was underpowered with a relatively small sample of patients recruited in this low risk population (stable CAD scheduled for PCI with warfarin stopped three days before PCI). Third, only AF patients were considered as eligible. It is uncertain if PRI values should be used in all TAT -treated subjects. Fourth, the only anticoagulant treatment prescribed was VKA. The authors did not evaluate the effects of the new DOACs. Fifth, the duration of TAT therapy was a fixed duration of three months (instead of six months in our study) followed by a fixed duration of DAPT with VKA.

Conversely, our study was conducted in real life, with VKAs agents and DOACs prescribed in a high-risk population (ACS patients, half of them were on HTPR for the ischemic risk, CHA_2_DS_2_VASc score 4.72 for the embolism risk and HAS-BLED score between 2 and 3 for bleeding risk). In addition, more than 50% of the patients in our study were treated with DOACs, and the median duration of TAT was 3.51 months which is quite different from Hu’s study [24]. Our results are consistent with recent RCTs with a 17% reported rate of bleeding events [3]. In our work, half of patients had HTPR, and numerous studies have shown that up to 40% of patients receiving clopidogrel developed HTPR [3].

In RCT, bleeding events were related to the duration and intensity of antiplatelet therapy [3] in DAPT. In our series, most bleeding events occurred within the first month because of INR imbalance issues. Ischemic events risk is increased within the first months after ACS as described in the AF-PCI trial. The high rate of MACCE early after PCI as observed in our work was confirmed in recent studies [3]. Our study confirms that DOACs showed the most favorable safety profile in TAT patients in comparison with VKA use. In our study more than half of MACCE and major bleeding occurred in the VKA group whether patient had HTPR or LTPR. In agreement with RCT trials, our study showed that DOAC seems to have a favorable efficacy risk profile with significant reduction in intracranial hemorrhages [25].

The recent nationwide Danish cohort study was unable to ascertain the clinical benefit of DOACs use because TAT regimen with VKA or DOAC was only prescribed in 1% and 0.3% of patients, respectively. However, further studies are needed to ascertain the superiority of DOAC over VKA in TAT regimen. Our observational data suggest that the incidence of serious anticoagulant related gastrointestinal bleeding is higher for rivaroxaban than for dabigatran and apixaban. This is consistent with previous studies [26,27]. One explanation may be that rivaroxaban is given once daily and the relative peak plasma concentrations are greater than those for other OAC [28]. When choosing OAC in TAT regimen, practitioners should assess the risk of gastrointestinal bleeding. In contemporary practice, our study as with the others RCT trials confirmed that MACCE are five times less common than bleeding occurrences [3]. The higher incidence of MACCE in our VKA group may be explained by the interaction between VKA and clopidogrel. It has been shown than clopidogrel and VKA agents cause an intra-drug interaction resulting in an attenuated antiplatelet effect of clopidogrel. VKA significantly alters the in vivo biotransformation of clopidogrel into its active thiol metabolite [19]. Our results are consistent with this observation. Among the 201 patients on VKA, the mean VASP index was 55.31% in VKA agents versus 44.62% in the DOAC group (data not shown). According with the guidelines, lower-dose rivaroxaban regimen (15 mg a day) was used and this may explain the increase of MACCE comparing with full-dose regimen of apixaban and dabigatran [3].

### 4.3. Strength and Limitation

The study is underpowered, as only 38 ischemic events were identified and the majority of these were all-cause deaths (*n* = 18, i.e., 47% of overall ischemic events).

This subject seems out of date because TAT has been shortened to one month and DOACs are strongly recommended [3]. Nevertheless, still many patients represent a challenge for interventional cardiologist because they present a high ischemic risk post PTCA (left main disease, history of stent thrombosis, etc.) and require several months of TAT [3]. Some practitioners continue to use PFT to guide de-escalation. This article should warn them about this falsely reassuring practice.

In our series, patients have a particularly severe profile when compared to the literature: older (mean age 78 years old), elevated CHA_2_DS_2_VASc score and high proportion of comorbidities. In addition, almost 60% of patients were treated with DOACs, and the median duration of TAT was 3.5 months, resulting in important differences with the data in the literature [1,2,3,4,5,6,24].

Many potential factors including clinical variables such as diabetes mellitus, renal failure, drug interactions and non-adherence to treatment may influence ischemic risk in ACS patients on TAT with clopidogrel due to its heterogeneous and unpredictable antiplatelet effect [3]. Genetic polymorphism of genes coding for clopidogrel absorption in intestines exert a crucial role in clopidogrel bioactivation. Clopidogrel is a prodrug, and ATP-binding cassette sub-family B member 1 (ABCB1) is involved in the intestinal absorption of the drug [29,30].

The optimal timing of testing with relationship to the PCI procedure remains a topic of debate. As for other biomarkers in cardio-vascular medicine such as troponins and pro–brain natriuretic peptide, a single test is a representative snapshot of the status quo for the time point when it is determined [22]. In addition, platelet reactivity is enhanced in the earlier phases of ACS and diminishes over time [22].

One strategy to resolve the question as to whether this genetic variation is involved in outcomes might be the method of Mendelian randomization.

Despite all these limitations, this is the first and the largest cohort of ACS patients on TAT regimen with PRI assessment in the real world. The sample of patients was close to the sub-groups in RCT, but in a high-risk population with every OAC (including DOAC) in all TAT-treated subjects (including mechanical heart valves and venous thromboembolism).

## 5. Conclusions

The optimal antithrombotic therapy after PCI in patients receiving long-term OAC remains controversial. RCTs have demonstrated that dual therapy (DOAC plus clopidogrel) significantly reduces bleeding risk in comparison to TAT regimen with non-significant trends towards higher risk of MI. Data were thus needed to help decision making to implement de-escalation strategy from TAT to dual therapy or to shorten the TAT regimen duration.

The use of VASP assays has been suggested to be a useful tool to help clinical decision, but our study, from the largest sample size to date, does not support this practice. However, our findings strongly suggest that DOAC should be prioritized.

## Figures and Tables

**Table 1:** Baseline characteristics for patients with six months follow-up (*N* = 491).

Variable	*N* = 491 (%)
Female	137 (28)
Age, year	78.4
Hypertension	353 (72)
Dyslipidemia	391 (79)
Smoker	147 (30)
Diabetes	
None	300 (60.8)
T1DM	24 (4.8)
T2DM	167 (34)
History of vascular disease	436 (89)
History of heart failure	197 (40.1)
History of hemorrhage	99 (20.1)
None	392 (80)
ENT	17 (3.4)
Cerebral	4 (0.8)
Digestive	37 (7.5)
Urinary	11 (2.2)
Hematoma	19 (3.8)
Vascular	11 (2.2)
History of ischemic stroke	34 (6.9)
History of CAD	207 (42.2)
None	284 (57.8)
MI	167 (34)
MI + Ischemic stroke	6 (1.2)
ACS	
STEMI	129 (26.3)
NSTEMI	324 (66)
Unstable Angina	38 (7.7)
LVEF, %	43.03
Indication for anticoagulation	
History of AF	372 (75.7)
New onset AF	77 (15.7)
VTED	24 (4.8)
Intracardiac thrombus	4 (0.8)
Coronary embolism	3 (0.6)
History of AF and VTED	6 (1.3)
Mechanical valve	5 (0.1)
GFR, mL/min	61.3 (21–96)
CHA_2_DS_2_VASc	4.7 (4–6)
HAS-BLED	2.5 (2–3)
Hemoglobin, mg/dL	128.8 (120–142)

ACS: Acute coronary syndrome; CAD: Coronary artery disease; ENT: Ear, nose, throat; LVEF: Left ventricular ejection fraction; MI: Myocardial infarction; NSTEMI: Non ST- elevation myocardial infarction; T1DM: Type 1 diabetes mellitus; T2DM: Type 2 diabetes mellitus; STEMI: ST-elevation myocardial infarction; VTED: Venous thromboembolism disease; AF: atrial fibrillation; GFR: Glomerular filtration rate; HAS-BLED: Hypertension, abnormal liver/renal function, stroke history, bleeding history or predisposition, labile INR, elderly, drug/alcohol usage.

**Table 2:** Indication for anticoagulation, major adverse cardiac or cerebrovascular events and bleedings for patients with six months follow-up (*N* = 491).

Variable	*N* = 491 (%)
Indication for anticoagulation	
History of AF	372 (75.7)
New onset AF	77 (15.7)
VTED	24 (4.8)
Intracardiac thrombus	4 (0.8)
Coronary embolism	3 (0.6)
History of AF and VTED	6 (1.3)
Mechanical valve	5 (0.1)
Triple therapy: aspirin+ clopidogrel associated with	
VKA	201 (41)
Rivaroxaban	112 (22.8)
Apixaban	108 (22)
Dabigatran	70 (14.2)
VASP, %	49 (25–69)
INR dosage	N
Under dosing INR	111 (55)
Targeted INR	80 (40)
Over-dosing INR	10 (5)
Length of triple therapy in months	3.51 (1–6)
PPI during triple therapy	455 (92.6)
Revascularization	
No PCI	2 (0.4)
PCI + stent	487 (99.2)
PCI without stent	2 (0.4)
Follow-up, days	190
MACCE with triple therapy	38 (7.7)
None	453 (92.2)
Non-fatal MI	16 (3.2)
Ischemic stroke	2 (0.5)
Death	18 (3.6)
Urgent revascularization	2 (0.5)
MACCE on TAT regimen	38 (7.7)
VKA	18 (3.6)
Rivaroxaban	14 (2.8)
Apixaban	3 (0.6)
Dabigatran	3 (0.6)
MACCE on rivaroxaban	14 (2.6)
Non-fatal MI	8 (1.6)
Death	4 (0.8)
Ischemic stroke	0 (0)
Urgent revascularization	2 (0.4)
MACCE on apixaban	3 (0.6)
Non-fatal MI	3 (0.6)
Death	0 (0)
Ischemic stroke	0 (0)
Urgent revascularization	0 (0)
MACCE on dabigatran	3 (0.6)
Non-fatal MI	3 (0.6)
Death	0 (0)
Ischemic stroke	0 (0)
Urgent revascularization	0 (0)
MACCE on VKA	18 (3.7)
Non-fatal MI	2 (0.4)
Death	14 (2.8)
Ischemic stroke	2 (0.4)
Urgent revascularization	0 (0)
Bleeding with triple therapy	85 (17.3)
None	406 (82.2)
Cerebral	4 (0.8)
VKA	2 (0.4)
Rivaroxaban	1 (0.2)
Dabigatran	1 (0.2)
Apixaban	0 (0)
Digestive	38 (7.8)
VKA	14 (2.8)
Rivaroxaban	18 (3.6)
Apixaban	4 (0.8)
Dabigatran	2 (0.4)
Hemoptysis	7 (1.5)
VKA	3 (0.6)
Rivaroxaban	1 (0.2)
Apixaban	1 (0.2)
Dabigatran	2 (0.2)
Urinary	11 (2.3)
VKA	9 (1.8)
Rivaroxaban	0 (0)
Apixaban	1 (0.2)
Dabigatran	1 (0.2)
Hematoma	13 (2.6)
VKA	10 (2)
Rivaroxaban	1 (0.2)
Apixaban	1 (0.2)
Dabigatran	1 (0.2)
Vascular	10 (2)
VKA	4 (0.8)
Rivaroxaban	1 (0.2)
Apixaban	0 (0)
Dabigatran	5 (1.0)
ENT	2 (0.4)
VKA	0 (0)
Rivaroxaban	0 (0)
Apixaban	0 (0)
Dabigatran	2 (0.4)
TIMI Class	85 (17.3)
Major	6 (1.2)
Minor	40 (8.1)
Minimal	39 (7.9)
Bleeding on TT regimen	85 (17.3)
VKA	42 (8.5)
Major	4 (0.8)
Minor	20 (4)
Minimal	18 (3.7)
Rivaroxaban	22 (4.5)
Major	2 (0.4)
Minor	15 (3.1)
Minimal	5 (1)
Apixaban	7 (1.8)
Major	0 (0)
Minor	3 (0.6)
Minimal	4 (1.2)
Dabigatran	12 (2.4)
Major	0 (0)
Minor	2 (0.4)
Minimal	12 (2)

AF: Atrial fibrillation; INR: International normalized ratio; MACCE: Major adverse cardiac and cerebrovascular events; MI: Myocardial infarction; PCI: Percutaneous coronary intervention; PPI: proton pump inhibitor; VASP: Vasodilator-stimulated phosphoprotein; TIMI: Thrombolysis in myocardial infarction; VKA: Vitamin K antagonist; VTED: Venous thromboembolism disease; TT: Triple therapy.

**Table 3:** Baseline characteristics for patients with 6 months follow-up, VASP cut-off 50% (N = 491).

Variable	VASP ≤ 50% *N* = 241	VASP > 50% *N* = 250	*p*
Female	76 (32)	61 (24)	NS
Age, year	79	78	NS
Hypertension	170 (70.5)	183 (73)	NS
Dyslipidemia	185 (76)	206 (82)	NS
Smoker	69 (28)	78 (32)	NS
Diabetes			
None	137 (56)	163 (65)	0.037
T1DM	17 (7)	7 (3)	
T2DM	87 (36)	80 (31)	
History of vascular disease	210 (87)	226 (90)	NS
History of heart failure	85 (35)	112 (44)	NS
History of hemorrhage	44 (18.2)	55 (22)	NS
ENT	6 (2.4)	11 (4.4)	
Cerebral	2 (0.8)	2 (0.8)	
Digestive	20 (8.2)	17 (6.8)	
Urinary	4 (1.7)	7 (2.8)	
Hematoma	8 (3.3)	11 (4.4)	
Vascular	4 (1.6)	7 (2.8)	
History of ischemic stroke	11 (44)	23 (9.2)	0.043
History of CAD	104 (43)	103 (42)	NS
None	137 (57)	147 (58)	
MI	90 (37)	77 (30.8)	
MI + Ischemic stroke	3 (1.2)	3 (1.2)	
ACS			NS
STEMI	79 (32.7)	50 (20)	
NSTEMI	152 (63)	172 (68.2)	
Unstable Angina	10 (4.1)	28 (11.2)	
LVEF, %	41 (35–59)	45 (35–60)	NS
Indication for anticoagulation			NS
History of AF	185 (76)	187 (75)	
Newonset AF	45 (18.6)	32 (12.8)	
VTED	5 (2)	19 (7.6)	
Intracardiac thrombus	1 (0.4)	3 (1.2)	
Coronary embolism	1 (0.4)	2 (1)	
History of AF and VTED	1 (0.4)	3 (2)	
Mechanical valve	3 (1.2)	2 (1)	
GFR, mL/min	56.88	62.09	0.016
CHA_2_DS_2_VASc	5.3 (4–6)	4.4 (4–5)	NS
HAS-BLED	2.6 (2–3)	2.45 (2–3)	NS
Hemoglobin, mg/dL	123 (121–140)	135 (120–143)	NS

VASP: Vasodilator-stimulated phosphoprotein; ACS: Acute coronary syndrome; CAD: Coronary artery disease; ENT: Ear, nose, throat; LVEF: Left ventricular ejection fraction; MI: Myocardial infarction; NSTEMI: Non ST- elevation myocardial infarction; T1DM: Type 1 diabetes mellitus; T2DM: Type 2 diabetes mellitus; STEMI: ST-elevation myocardial infarction; VTED: Venous thromboembolism disease; GFR: Glomerular filtration rate; HAS-BLED: Hypertension, abnormal liver/renal function, stroke history, bleeding history or predisposition, labile INR, elderly, drug/alcohol usage.

**Table 4:** Indication for anticoagulation, major adverse cardiac or cerebrovascular events and bleeding for patients with six months follow-up, VASP cut-off 50% (*N* = 491).

Variable	VASP ≤ 50% *N* = 241	VASP > 50% *N* = 250	*p*
Triple therapy: aspirin+ clopidogrel associated with			
VKA	110 (45.6)	91 (36.4)	
Rivaroxaban	52 (21.6)	60 (24)	
Apixaban	48 (19.9)	60 (24)	
Dabigatran	31 (13.3)	39 (15.2)	
VASP, %	27.5 (22–37)	75 (64–76)	<0.001
INR dosage			NS
Under dosing INR	51 (50)	60 (59)	
Targeted INR	51 (44)	39 (35)	
Over-dosing INR	9 (8)	1 (1)	
Length of triple therapy in months	3.5 (1–6)	3.6 (1–6)	NS
PPI during triple therapy	230 (95)	225 (90)	NS
Revascularization			NS
No PCI	0 (0)	2 (0.4)	
PCI + stent	241 (100)	246 (98.4)	
PCI without stent	0 (0)	2 (0.8)	
Follow-up, days	190 (190–190)	190 (190–190)	NS
MACCE with triple therapy	23 (9.5)	15 (6)	NS
Non-fatal MI	9 (3.7)	7 (2.8)	
Ischemic stroke	1 (0.4)	1 (0.4)	
Death	11 (4.6)	7 (2.8)	
Urgent revascularization	2 (0.8)	0 (0)	
MACCE on TAT regimen	23 (9.5)	15 (6)	NS
VKA	10 (4.1)	8 (3.2)	
Rivaroxaban	10 (4.1)	4 (1.6)	
Apixaban	1 (0.4)	2 (0.8)	
Dabigatran	2 (0.8)	1 (0.4)	
Bleeding with triple therapy	33 (13.6)	52 (20.8)	0,04
Cerebral	2 (0.8)	2 (0.8)	
Digestive	12 (5)	26 (10.4)	
Hemoptysis	3 (1.2)	4 (1.6)	
Urinary	2 (0.8)	9 (3.6)	
Hematoma	11 (4.6)	2 (0.8)	
Vascular	1 (0.4)	9 (3.6)	
ENT	2 (0.8)	0 (0)	
TIMI class	33 (13.6)	52 (20.8)	0.04
Major	3 (1.2)	3 (1.2)	
Minor	15 (6.2)	25 (10)	
Minimal	15 (6.2)	24 (10)	
Bleeding on TAT regimen	33 (13.6)	52 (20.8)	0.04
VKA	19 (7.8)	23 (9.2)	
Rivaroxaban	11 (4.5)	11 (4.8)	
Apixaban	0 (1)	7 (2.4)	
Dabigatran	3 (0.5)	11 (4.4)	

AF: Atrial fibrillation; INR: International normalized ratio; MACCE: Major adverse cardiac and cerebrovascular events; MI: Myocardial infarction; PCI: Percutaneous coronary intervention; PPI: proton pump inhibitor; VASP: Vasodilator-stimulated phosphoprotein; TIMI: Thrombolysis in myocardial infarction; VKA: Vitamin K antagonist; VTED: Venous thromboembolism disease; TAT; triple antithrombotic therapy; ENT: Ear, nose, throat.

**Table 5:** Baseline characteristics for patients with six months follow-up, VASP cut-off 16% (*N* = 491).

Variable	VASP ≤ 16% *N* = 39	VASP > 16% *N* = 452	*p*
Female	12 (30.7)	125 (27.7)	NS
Age, year	79.1	78.3	NS
Hypertension	27 (69.2)	326 (72.1)	NS
Dyslipidemia	28 (71.7)	363 (80.3)	NS
Smoker	11 (28.2)	136 (30)	NS
Diabetes			NS
None	20 (51.2)	280 (61.9)	NS
T1DM	3 (7.7)	21 (4.6)	
T2DM	16 (41)	151 (34.7)	
History of vascular disease	31 (79)	405 (89.6)	NS
History of heart failure	12 (30.7)	185 (40.9)	NS
History of hemorrhage	10 (25)	89 (19)	NS
ENT	1 (2.5)	16 (3.5)	
Cerebral	2 (5)	2 (0.4)	
Digestive	2 (5)	35 (7.7)	
Urinary	2 (5)	9 (é)	
Hematoma	2 (5)	17 (3.è)	
Vascular	1 (2.5)	10 (2.2)	
History of ischemic stroke	5 (12)	29 (6.4)	NS
History of CAD	13 (33)	194 (43)	NS
None	26	258	
MI	07 (18)	160 (36)	
MI + Ischemic stroke	1 (3)	5 (1.1)	
ACS	39	452	NS
STEMI	15 (38.5)	114 (25.2)	
NSTEMI	20 (51.2)	304 (67.2)	
Unstable Angina	4 (10.2)	34 (7.5)	
LVEF, %	43.92 (35–59)	41.3 (35–60)	NS
GFR, mL/min	57.79	59.09	NS
CHA_2_DS_2_VASc	4.67 (4–5)	5.23 (4–6)	0.02
HAS-BLED	2.5 (2–3)	2.72 (2–3)	NS
Hemoglobin, mg/dL	127 (121–140)	131 (120–143)	NS

VASP: Vasodilator-stimulated phosphoprotein; ACS: Acute coronary syndrome; CAD: Coronary artery disease; ENT: Ear, nose, throat; LVE: Left ventricular ejection fraction; MI: Myocardial infarction; NSTEMI: Non ST- elevation myocardial infarction; T1DM: Type 1 diabetes mellitus; T2DM: Type 2 diabetes mellitus; STEMI: ST-elevation myocardial infarction; GFR: Glomerular filtration rate; HAS-BLED: Hypertension, abnormal liver/renal function, stroke history, bleeding history or predisposition, labile INR, elderly, drug/alcohol usage.

**Table 6:** Indication for anticoagulation, major adverse cardiac or cerebrovascular events and bleedings for patients with six months follow-up, VASP cut-off 16% (*N* = 491).

Variable	VASP ≤ 16 % *N* = 39	VASP > 16% *N* = 452	*p*
Triple therapy: aspirin+ clopidogrel associated with			0.002
VKA	13 (33.3)	188 (41.5)	
Rivaroxaban	7 (18)	105 (22.2)	
Apixaban	10 (25.6)	98 (21.7)	
Dabigatran	09 (23)	61 (13.5)	
VASP, %	9.5 (10–16)	53 (64–76)	<0.001
INR dosage			NS
Under dosing INR	5 (38.2)	106 (56.4)	
Targeted INR	4 (30.4)	76 (40.4)	
Over-dosing INR	4 (30.4)	6 (3.1)	
Length of triple therapy in months	3.72 (1–6)	3.58 (1–6)	NS
PPI during triple therapy	35 (89)	42 (93)	NS
Revascularization			NS
No PCI	1	1 (0.2)	
PCI + stent	37 (99)	450 (99.6)	
PCI without stent	1	1 (0.2)	
Follow-up, days	190 (190–190)	190 (190–190)	NS
MACCE with triple therapy	4 (10.2)	34 (7.6)	NS
Non-fatal MI	1 (2.5)	15 (3.3)	
Ischemic stroke	1 (2.5)	1 (0.30)	
Death	1 (2.5)	17 (3.7)	
Fatal MI	1 (2.5)	1 (0.3)	
MACCE on TT regimen	4 (10.2)	34 (7.6)	NS
VKA	2 (5.1)	16 (3.5)	
Rivaroxaban	2 (5.1)	12 (1.8)	
Apixaban	0 (0)	3 (0.7)	
Dabigatran	0 (0)	3 (1.5)	
Bleeding with triple therapy	6 (15.4)	79 (17.5)	NS
Cerebral	1 (2.5)	3 (0.4)	
Digestive	3 (7.6)	35 (7.9)	
Hemoptysis	0	7 (1.5)	
Urinary	0	11 (2.4)	
Hematoma	0	13 (2.8)	
Vascular	1 (2.5)	9 (1.9)	
ENT	1 (2.5)	1 (0.2)	
TIMI class	*N* = 6	*N* = 79	NS
Major	2 (5.1)	4 (0.8)	
Minor	2 (5.1)	38 (8.5)	
Minimal	2 (5.1)	37 (8.2)	
Bleeding on TT regimen		40	NS
VKA	2	20	
Rivaroxaban	2	8	
Apixaban	1	11	
Dabigatran	1		

VASP: Vasodilator-stimulated phosphoprotein; AF: Atrial fibrillation; INR: International normalized ratio; MACCE: Major adverse cardiac and cerebrovascular events; MI: Myocardial infarction; PCI: Percutaneous coronary intervention; PPI: proton pump inhibitor; VASP: Vasodilator-stimulated phosphoprotein; TIMI: Thrombolysis in myocardial infarction; VKA: Vitamin K antagonist; VTED: Venous thromboembolism disease; TT: Triple therapy.

## Data Availability

The data presented in this study are available on request from the corresponding author.
